# The State Board of Health

**Published:** 1884-07

**Authors:** 


### Editorial.

### THE STATE BOARD OF HEALTH.

Plutarch, in his curious work on Morals, upon the authority of a much earlier author, Aristodemus, relates that in the time of a great plague in Lacedæmon, the people were told by the oracle that the pestilence would cease upon sacrificing a noble virgin every year. The victim was selected by lot, and the horrible hygienic expedient became common and was long practiced. In the presence of a great calamity, in the midst of a wide-spread and fatal epidemic, public authority will hurriedly adopt any measures, however absurd, inconvenient or costly, whether proposed by the skilled or the ignorant, and public approval will in moments of alarm sustain and enforce them. "But it is human nature to soon forget past sufferings. We bury our dead; a little time will dry our tears; in another little time we dance over their graves." Experience has shown the extreme difficulty of persistently maintaining any regulations for the preservation of the public health. The danger to be guarded against is intermittent, remote, uncertain; vigilance relaxes, continued restraints fatigue, and the wisest precautions are disused or neglected. This difficulty is increased by the fact, that often the measures proposed are inefficient or framed in ignorance of, or in violation of scientific truths, and command no intelligent approval. The sacrificial offering of a virgin or any other merely superstitious observance would not in this age be publicly proposed, however it might be privately practiced, but general and local legislation furnish too many instances of misguided action based upon merely speculative opinions not generally credited. These, of course, are quickly disregarded and consigned to oblivion, the great receptacle of the old and the new speculative philosophy.

All preventive medicine must be based as nearly as possible upon exact knowledge of the causes of disease. These, coming from so many sources—from the air, the earth, the water, from all physical agencies, from the quantity and quality of food and drink, from the personal and social habits of mankind, from mysterious and unknown poisonous agencies occasionally widely diffused—demand an extent of observation and painstaking experiment which must render the progress of the science extremely slow. Centuries may elapse before it reaches perfection. Up to the point which it has or may attain, legislation should encourage and sustain it. Every step of State Medicine should be guided by the hand of the scientific expert; it can stand securely alone upon the solid rock of ascertained facts, but all advance into the region of doubt and uncertainty fails to carry with it public confidence and results in disappointment.

The unaided efforts of private physicians have accomplished much, but in so difficult a science, eminence in it which would entitle one to be regarded as an expert, would demand a devotion impossible amid the varied cares of professional life. Adequate reward would soon develop a class of scientists able to secure to the public the full benefit of all that is known, and continually add to the present accumulations by new discoveries.

The establishment of boards of health, sanitary commissions and other like bodies in very many communities, is the recognition by the Legislatures of the necessity of movement in this direction. These bodies when in existence are doubtless faithfully carrying out the objects of their institution, to the extent of their means and authority; good results will demonstrate the propriety of an increase of both. Reports from many of them published from time to time are encouraging, and the history of them all would be instructive.

In the interest of preventive medicine the General Assembly of the State of Georgia, at the suggestion of the State Medical Association, in 1875, passed an act to create a State Board of Health. The act required the Governor to appoint nine physicians of skill and experience, who shall have been regular graduates of medicine and practitioners of not less than ten years, who, together with the Comptroller-General, Attorney-General and State Geologist, shall constitute the Board of Health of the State of Georgia.

The Governor organized the Board by the appointment of nine gentlemen who came fully up to the requirements of the law. Indeed he seems to have been fortunate in his selections. They were all physicians of established reputation and favorably known in the respective sections of the State in which they were required to reside, and with professional zeal, patriotically entered upon the discharge of their prescribed duties. These duties are generally enumerated in the fifth and sixth sections of the act, as follows:

SECTION V. *Be it further enacted*, That said Board shall take cognizance of the interest of health and life among the people of the State; they shall make inquiries in respect to the causes of diseases, and especially of epidemics, and investigate the sources of mortality, and the effects of localities, employments, and other conditions upon the public health.

SEC. VI. *Be it further enacted*, That it shall be the duty of said Board to obtain, collect and preserve such information relating to deaths, diseases and health, as may be useful in the discharge of its duties, and contribute to the promotion of health, or the security of life in the State of Georgia; and it shall be the duty of all health officers, and Boards of Health in the State, to communicate to said State Board of Health copies of all their reports and publications; also, such sanitary information as may be useful, and said Board shall keep record of its acts and proceedings as a Board, and it shall promptly cause all proper information in possession of said Board to be sent to the local health authorities of any city, village, or town in the State which may request the same, and shall add thereto such useful suggestions as the experience of said Board may supply; and it is hereby made the duty of said health authorities to supply like information and suggestions to said State Board of Health, and said State Board of Health is authorized to require reports and information (at such times, and of such facts, and generally of such nature and extent, relating to the safety of life and the promotion of health, as its by-laws or rules may provide) from all public dispensaries, hospitals, asylums, prisons and schools, and from the managers, principals and officers thereof; and from all other public institutions, their officers and managers, and from the proprietors, managers and lessees and occupants of all places of public resort in the State; but such reports and information shall only be required concerning matters or particulars in respect of which it may, in its opinion, need information for the proper discharge of its duties. Said Board shall, when requested by public authorities, or when they deem it best, advise officers of the State, county or local governments in regard to sanitary drainage, and the location, drainage, ventilation and sanitary provisions of any public institution, building or public place.

Another section of the act required an annual report in writing to be laid before the General Assembly upon the sanitary condition and prospects of the State, and that such report shall set forth the action of said Board, its officers and agents, and may contain other useful information, and shall suggest any further legislation, action or precaution deemed proper for the better protection of life and health.

It would be difficult more wisely to constitute a State sanitary commission, or more clearly to declare its objects and purposes. The magnificent possibilities of its faithfully prosecuted labors are obvious to every intelligent mind, but unfortunately the law contained two important defects. In subsequent sections it required the establishment of, and burdened the Board of Health with the supervision, of a State system of registration of births, marriages and deaths, and the superintendence of a registration of vital statistics. The details of this system imposed upon the ordinaries and coroners of the respective counties, upon physicians and upon the whole body of citizens, unaccustomed formalities and duties which they deemed burdensome or useless, and notwithstanding they were enforced by heavy penalties, they were disregarded and could not be generally carried into effect.

Another fatal defect of the law was that it provided no compensation for the commissioners, in fact, prohibited all salary. They were expected to perform all the multifarious duties of their position, requiring the highest scientific attainments, great labor, sacrifice of time and exposure to all pestilence, gratuitously. The appropriation to sustain this important institution was limited to fifteen hundred dollars, one thousand for clerical services, leaving five hundred (a sum totally inadequate) for the collection and publication of information in relation to all subjects in the line of their inquiries. These imperfections in the law were manifest, but in the hope that the Legislature would from time to time perfect it, upon the suggestions they were required to make in their annua reports, the "sanitary commission" began its labors. No one has questioned the zeal or intelligence with which they attempted the execution of their thankless and unrewarded task, but the greater their activity, the stronger became the popular opposition, and very soon the Legislature cut off the small appropriation on which the continuance of the experiment depended.

No body of men, except the Christian ministry, are willing to perform as much unrequited toil as physicians; but the maintenance of the Board of Health at their personal charge was too heavy a burden on the Sanitary Commissioners, and they ceased to meet. The law was not repealed, it simply fell into desuetude.

The recognized importance of preventive medicine which prompted the creation of the Board of Health is undiminished. Interest in the subject is increasing throughout the civilized world. Communities everywhere have established like bodies having similar powers and purposes. Undiscouraged by a single failure, the friends of scientific progress in Georgia desire to renew the attempt and to keep this State and community abreast with the advancing thought and progress of the age.

The time and manner of doing so are undetermined. The duty devolves upon the medical profession, and its importance solicits their wisest consideration.

At first view it would seem that the reorganization of the Medical Board as created by the law of 1875 would be simple and easy, trusting to future legislation to amend and perfect it, but examination shows that although that law,intended to be of perpetual obligation has never been repealed, it has somehow ceased to be of force, and any State action must be begun *de novo.*

How this conclusion was reached will, it is hoped, amuse, if it does not instruct, the readers of this journal. Able lawyers are at stated times appointed to codify all the laws of the State. It was alleged that in the last code certain laws had been omitted, and among others that referred to above, creating the Board of Health, and also another creating a Geological Department, the chief of which was also a member of the Board of Health.

Counsel learned in the law was employed (it is hoped at honorable compensation) to report upon what legislation it omits, that should have been inserted. It is needless to say that the Legislature wisely retained for this service an acknowledged expert, one whose eminence was so unquestioned that like Job of the olden time "after he had spoken no man opened his mouth." Below is appended that portion of the report which relates to

"*The State as patron and beneficiary of Applied Science—scientific observation, research and elaboration—Geological department—Board of Health.*

At present both these institutions are (to say the least) in a state of legal torpor or suspended animation. The charters of their nativity, the statutes under which they were born, being unrepealed, they are still under the "old flag," but they lie in a protracted swoon for lack of an 'appropriation." Moreover, as to the former, there has been a grant of administration upon its assets, which grant could only have been made upon the assumption that it was defunct. (Session Laws, 1878-79, p 434.) And this assumption has been followed by a peculiar *post mortem* resolution, a sort of capital sentence, *nunc pro tunc*—a judgment of death pronounced after execution. (Session Laws, 1880-1, p. 696.) In the presence of documents apparently so ultimate, so final, it is not easy to accept for good law the teachings of the Code in §1465d, that the Commissioner of Agriculture is entitled to employ a geologist to assist him in preparing a geological survey, or the still more apocryphal statement, a little further on, to the effect that there is a standing appropriation of ten thousand dollars a year to pay the expenses of such survey, together with the other enumerated objects of annual expenditure. If these provisions of the Code touching geological possibilities are sound considerably more on the same subject might have been, perhaps ought to have been, introduced. On practical grounds, however, I am persuaded that it would have been safe to have left

The pan mortuary hypothesis is the one which has the sanction of official practice. I see not how it could be contested with any advantageous results. What can justly and forcibly be said is, that the dying at Board headquarters has been informal and irregular, but it is none the less real; and I am not aware that any vital interest of the departed, whether legal or sentimental, can be served by mere protest against the manner of the "taking off." There is not in law, any more than in nature, a doctrine that death is void for irregularity.

